# Combining Immunotherapy and Radiotherapy for Cancer Treatment: Current Challenges and Future Directions

**DOI:** 10.3389/fphar.2018.00185

**Published:** 2018-03-05

**Authors:** Yifan Wang, Weiye Deng, Nan Li, Shinya Neri, Amrish Sharma, Wen Jiang, Steven H. Lin

**Affiliations:** ^1^Department of Experimental Radiation Oncology, The University of Texas MD Anderson Cancer Center, Houston, TX, United States; ^2^The University of Texas MD Anderson Cancer Center UTHealth Graduate School of Biomedical Sciences, Houston, TX, United States; ^3^Department of Radiation Oncology, The University of Texas MD Anderson Cancer Center, Houston, TX, United States

**Keywords:** radiotherapy, immunotherapy, immune checkpoints, cancer treatment, biomarkers

## Abstract

Since the approval of anti-CTLA4 therapy (ipilimumab) for late-stage melanoma in 2011, the development of anticancer immunotherapy agents has thrived. The success of many immune-checkpoint inhibitors has drastically changed the landscape of cancer treatment. For some types of cancer, monotherapy for targeting immune checkpoint pathways has proven more effective than traditional therapies, and combining immunotherapy with current treatment strategies may yield even better outcomes. Numerous preclinical studies have suggested that combining immunotherapy with radiotherapy could be a promising strategy for synergistic enhancement of treatment efficacy. Radiation delivered to the tumor site affects both tumor cells and surrounding stromal cells. Radiation-induced cancer cell damage exposes tumor-specific antigens that make them visible to immune surveillance and promotes the priming and activation of cytotoxic T cells. Radiation-induced modulation of the tumor microenvironment may also facilitate the recruitment and infiltration of immune cells. This unique relationship is the rationale for combining radiation with immune checkpoint blockade. Enhanced tumor recognition and immune cell targeting with checkpoint blockade may unleash the immune system to eliminate the cancer cells. However, challenges remain to be addressed to maximize the efficacy of this promising combination. Here we summarize the mechanisms of radiation and immune system interaction, and we discuss current challenges in radiation and immune checkpoint blockade therapy and possible future approaches to boost this combination.

## Introduction

The success of immunotherapy in treating some form of cancer has greatly encouraged researchers and clinicians to combine it with other conventional therapies to improve effectiveness still further. One such therapy, radiation, is commonly used to treat many types of cancer, and its combination with immunotherapy is considered promising. This combination is expected to have synergistic effects stemming from both local and systemic tumor control due to the unique and intriguing interactions between radiation and the immune system (Jiang et al., 2016; Frey et al., 2017; Son et al., 2017). Radiation's local therapeutic effects result from direct damage to cancer cells causing cell death and from triggering activation of CD8^+^ T cells (Lee et al., 2009). On the other hand, the systemic immune response can also be triggered by radiation-induced microenvironmental changes to tumor cells as well as the surrounding stromal cells (Jiang et al., 2016). Thus, the combination of radiation and immunotherapy could be more potent than either treatment alone, as has been shown in preclinical models (Deng et al., 2014a; Dovedi et al., 2014; Twyman-Saint Victor et al., 2015). The benefits of combining radiation and immunotherapy have been reported in several case reports for different cancer types, including head and neck squamous cell carcinoma (Nagasaka et al., 2016), metastatic pancreatic cancer (Shi et al., 2017), metastatic melanoma (Haymaker et al., 2017), lung cancer (Schoenhals et al., 2016), and brain metastases (Alomari et al., 2016). Currently, there are numerous ongoing clinical trials testing the combination of immunotherapy and radiation (Kang et al., 2016; Kumar S. S. et al., 2017; Weichselbaum et al., 2017). Recent results of the phase III randomized trial (PACIFIC) testing the role of the PDL1 antibody durvalumab vs. placebo as consolidation therapy after chemoradiation for stage III non-small cell lung cancer (NSCLC) demonstrated substantial improvement in progression-free survival (PFS) with durvalumab (16.8 months vs. 5.6 months with placebo), with similar types and severity of side effects (Antonia et al., 2017). Although these results were impressive and will likely change the standard of care in stage III NSCLC, challenges remain for the future development of this combination therapy. Below we summarize the known mechanisms by which radiotherapy and immunotherapy interact, and we discuss precautions to take in the future and possible approaches to further boost the effectiveness of this combination.

## Mechanistic rationale for combining radiation with immunotherapy

### Radiation increases antigen visibility

During the development of cancer, the relationship between the tumor and the host immune system evolves from one in which the tumor cells are recognized and destroyed by the immune system (immune elimination) to immune equilibrium, where tumor cells and immune system coexist, and finally to immune escape. The immune escape stage is characterized by upregulated inhibitory ligands and cytokines, reduced MHC class I expression, and increased numbers of myeloid-derived suppressor cells (Kalbasi et al., 2013). This overall immunosuppressive environment causes poor antigen presentation and masks the tumor from immune surveillance and elimination. However, radiation may “unmask” the tumor, making it visible to both the innate and adaptive immune systems (Jiang et al., 2016). The first step in this process is the activation of downstream immune responses and priming of T cells, which requires that antigen-presenting cells engulf the tumor cells and present their antigens to naïve T cells through phagocytosis. The presence of the calcium-binding protein calreticulin is a key signal to promote phagocytosis (Obeid et al., 2007). In one study, targeting HER2-positive tumors with a multivalent bi-specific nanobioconjugate engager conjugated with calreticulin protein increased phagocytosis of tumor cells by macrophages and enhanced the priming of T cells (Yuan et al., 2017). Radiation seems to promote the translocation of calreticulin from the endoplasmic reticulum to the plasma membrane (Golden et al., 2012). Meanwhile, the protein that triggers the anti-phagocytosis signal CD47 may be downregulated upon radiation exposure (Vermeer et al., 2013). Another key factor modulating activation of an immune response, high mobility group box 1 (HMGB1), is released from tumor cells upon exposure to x-ray or carbon-ion radiation (Yoshimoto et al., 2015). In short, radiation acts to enhance the clearance of damaged tumor cells by the antigen-presenting cells, thereby promoting the priming of T cells. Second, downregulation of MHC-I expression on tumor cells, typical of several types of cancer, causes poor recognition of those cancer cells by the cytotoxic T cells (Marincola et al., 2000). Radiation can upregulate the expression of MHC-I on the tumor surface to enable better presentation of tumor-specific peptides, enhancing the visibility of the tumor to cytotoxic T cells (Reits et al., 2006). By inducing a systemic increase in antigen recognition, radiation may also induce the T cell-mediated inhibition of untreated distant tumors (known as the abscopal effect) (Demaria et al., 2004). The ability to increase tumor antigen presentation also makes radiation a promising modality to be combined with Chimeric Antigen Receptor (CAR) T-cell therapies. CAR T-cells are considered as a “living drug,” since the therapy utilizes T cells isolated from patients and genetically engineers the T cells to express CAR that recognize tumor-specific antigens. Once infused back to patients, CAR T-cells are able to recognize tumor cells and induce cell death. Two CAR T-cell therapies have been approved for treating acute lymphoblastic leukemia and advanced lymphoma. However, using CAR T-cell therapies for solid tumors could be challenging due to difficulties in target selection (Flynn et al., 2017). Radiation could increase and MHC-I expression and tumor-specific antigens to make the tumors a more feasible target of CAR T-cells (Flynn et al., 2017). Third, radiation-induced DNA damage may generate neoantigen and trigger the immune surveillance. It is recently reported that DNA mismatch repair-deficient cancer cells grew poorly in immunocompetent mice but not in immunocompromised mice. The accumulated DNA mutations in cells with DNA repair deficiency increased the burden of neoantigens and triggered the immune response (Germano et al., 2017). It is plausible that the DNA damage induced during the course of radiotherapy may also enhance the mutational load and provide neoantigen for immune recognition, particularly in combination with DNA repair inhibitors.

However, the combination of radiotherapy with immunotherapy could be a double-edged sword. Since immune checkpoint blockade changes the equilibrium between immunity and tolerance, a higher rate of immune reaction with normal tissues accompanies with the increased likelihood of tumor recognition. Clinically, patients treated with immune checkpoint inhibitors could have immune-related adverse events (irAEs), such as fatigue, rash, skin disorders, colitis, and GI events (Alsaab et al., 2017; Kumar V. et al., 2017). When the tumor is treated by radiation, not only tumor-specific antigens but also non-tumor-specific antigens could be released into the tumor microenvironment. Some of the non-tumor-specific antigens might prime auto-reactive T cells which will attack and damage normal tissues if not properly negatively selected (Tang et al., 2018). Recent retrospective studies indicate the adverse events were increased when immunotherapies were combined with EGFR-TKI for NSCLC (Oshima et al., 2018) or with radiation for brain metastases (Martin et al., 2018). These findings warrant preclinical studies to investigate the biological mechanisms underlying the increased toxicity and potential methods to lower such risks. Future prospective clinical studies are needed to improve our understanding of the benefits and risks associated with such combinations.

### Radiation activates the cGAS-STING pathway to trigger immune responses

Radiation not only kills tumor cells directly but also seems to activate innate and adaptive immune responses through the Stimulator of Interferon Genes (STING) -mediated DNA-sensing pathway. STING is essential to protect hosts from DNA pathogens (Sharma et al., 2011; Watson et al., 2012). When the presence of cytoplasmic DNA is detected, the product of cyclic GMP-AMP synthase (cGAS), cyclic GMP-AMP (cGAMP), activates STING to upregulate transcription of a type I interferon gene through a STING-TBK-IRF3-NFκB signaling pathway (Ishikawa et al., 2009; Tanaka and Chen, 2012; Li et al., 2013; Sun et al., 2013). The STING pathway plays a critical role in anti-cancer immunity, as this pathway has been reported to be frequently lost in cancers including colorectal carcinoma and melanoma (Xia et al., 2016a,b). The STING pathway is essential for radiation-induced, type I interferon-dependent antitumor immunity (Deng et al., 2014b). Silencing of cGAS in bone marrow-derived dendritic cells was shown to impair their T-cell priming function when they were co-cultured with irradiated cells (Deng et al., 2014b). Because of the growing evidence of STING's critical role in anti-tumor immunity, STING agonists could be promising cancer therapeutics which have been investigated in preclinical and clinical studies. It has been demonstrated that direct STING activation by intra-tumoral administration of STING agonist resulted in both local and systemic anti-tumor immune response (Corrales et al., 2015). The combination of cyclic dinucleotides, a STING activator, with image-guided radiotherapy synergistically controlled both local and distant pancreatic cancer in a murine model (Baird et al., 2016). Currently, a STING agonist, MIW815 (ADU-S100), is under investigation in a phase I clinical trial (NCT02675439) to evaluate its safety and efficacy in patients with advanced solid tumors or lymphomas. However, much of the biologic mechanism of STING is still unknown. Despite numerous studies showing the immune stimulation function of STING, the role of STING pathway in anti-tumor immunity could be quite intriguing. It has been reported that STING deficiency protects mice from 7,12-dimethylbenz(a)anthracene induced skin cancer by decreasing inflammatory cytokine release (Ahn et al., 2014). The STING pathway activation may also enhance indoleamine 2,3 dioxygenase activity in the tumor microenvironment and induces immune tolerance in the lewis lung carcinoma model (Lemos et al., 2016). A recent study suggested that STING activation after radiation could drive immunosuppression. Radiation-induced STING and type I interferon activation recruits myeloid-derived suppressor cells to the irradiated tumor through the CCR2 pathway, causing immunosuppression and radioresistance (Liang et al., 2017). In addition, the mechanism of how DNA released from damaged cancer cells is transferred to antigen presenting cells to activate the STING pathway is still not clearly understood (Corrales et al., 2016). More studies into the biological mechanisms and the therapeutic potential of the STING pathway are still needed.

### Radiation modifies tumor stromal microenvironments

A tumor is not an isolated island of tumor cells, but a complex organ supported by stromal cells and blood vessels (Hanahan and Weinberg, 2011). Stromal cells and their secreted signals (cytokines, chemokines, and growth factors) constitute the major portion of the tumor microenvironment. Several cytokines can be induced by radiation (Barker et al., 2015), one of the most critical of which is transforming growth factor β (TGF-β). TGF-β signaling is upregulated momentarily after radiation (Klopp et al., 2007) and triggers an immune-suppressive microenvironment by reducing the cytotoxicity of CD8^+^ T cells, suppressing CD4^+^ T-cell differentiation, promoting regulatory T cell (Treg) transformation, and inhibiting natural killer (NK) cell proliferation (Trapani, 2005; Wrzesinski et al., 2007; Yang et al., 2010). TGF-β is also involved in the activation of vascular endothelial growth factor (VEGF) transcription (Liu et al., 2012). Given these immunosuppressive actions of TGF-β, attempts have been made to inhibit TGF-β signaling after radiation. In one preclinical study, radiation combined with TGF-β neutralization increased T cell priming and decreased tumor growth and metastasis (Vanpouille-Box et al., 2015), and the addition of anti-PD1 therapy to this combination further extended the survival of the experimental mice (Vanpouille-Box et al., 2015).

## Challenges in combining radiation and immunotherapy

### Optimizing the timing of radiotherapy and immunotherapy

**Figure 1:**
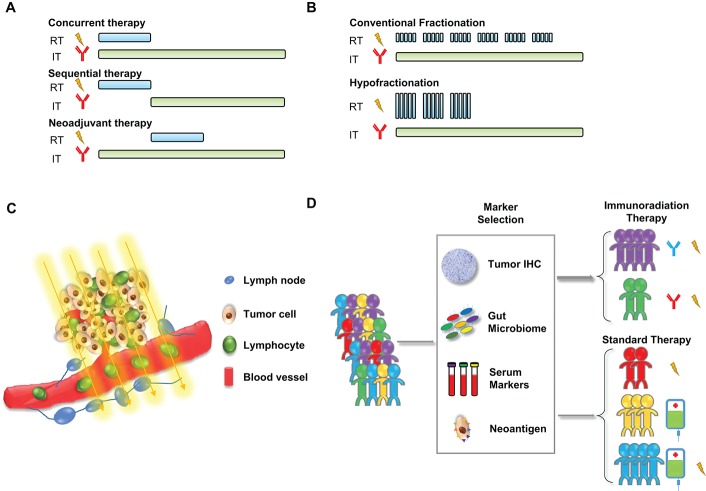
Current challenges in combining radiotherapy with immunotherapy. **(A)** Optimization of treatment timing: using immunotherapy concurrently, sequentially, or as neoadjuvant therapy with radiotherapy. **(B)** Optimization of radiation dosing: conventional fractionation or hypofractionation. **(C)** Reduction of the radiation-induced toxicity of circulating and tumor-infiltrated lymphocytes. **(D)** Selection of immunoradiation therapy or standard therapy for patients based on predictive biomarkers.

In any combination treatment that involves several treatment modalities, the timing of each component could be critical to the outcome. Since different types of immunotherapy target different pathways or different immune cells, the strategy of treatment combinations should be carefully designed to produce the greatest synergistic effects (Figure 1A). To date, several preclinical and clinical studies have been carried out to interrogate this question. So far, the results appear to suggest that the optimal timing is tumor type and immunotherapy-specific. In a mouse study testing combinations of hypofractionated radiation therapy (20 Gy) and immunotherapy drugs, anti-CTLA4 was found to work most effectively when given before the radiation, but anti-OX40 was more effective when given 1 day after the radiation (Young et al., 2016). A study of patients with melanoma brain metastases showed that concurrent immunotherapy with anti-PDL1 and anti-CTLA4 given within 4 weeks of stereotactic radiosurgery led to improved response of brain lesions relative to treatments that were separated by more than 4 weeks (Qian et al., 2016). A preclinical mouse study showed that resistance to fractionated radiotherapy could be overcome by PDL1 blockade, but PDL1 inhibition was effective only when given either concomitantly with or at the end of radiation, not 1 week following radiation (Dovedi et al., 2014). The secondary analysis of the KEYNOTE-001 trial (NCT01295827) showed the NSCLC patients who received radiotherapy before pembrolizumab (anti-PD1) had better overall survival and progression-free survival compared with the patients who did not receive radiotherapy (Shaverdian et al., 2017), suggesting radiation may enhance the efficacy of immunotherapy. Interestingly, analysis of the PACIFIC trial (Antonia et al., 2017) examining the timing when durvalumab was started relative to the completion of chemoradiotherapy suggests that starting durvalumab within 14 days after completing chemoradiotherapy appeared to have greater PFS efficacy than if durvalumab were started after 14 days. A recent retrospective review of 758 patients who received immunotherapy (anti-CTLA4 and/or anti-PD1/anti-PDL1) and radiotherapy suggested that overall survival was better for patients who received concurrent immunotherapy and radiotherapy (Samstein et al., 2017). Among the patients who received concurrent therapy, survival was longer when induction immunotherapy was begun more than 30 days before radiation compared with immunotherapy begun within 30 days before radiation (median overall survival times 20 vs. 11 months) (Samstein et al., 2017). Collectively, this preclinical and clinical evidence strongly suggests that the scheduling of radiotherapy and immunotherapy must be considered carefully, ideally in the context of clinical trials. One phase II trial currently ongoing at MD Anderson Cancer Center (NCT02525757) that considers the timing of immunotherapy is evaluating the safety and efficacy of atezolizumab, a monoclonal antibody targeting PDL1, in combination with standard chemoradiation (carboplatin and paclitaxel plus conventional 2-Gy fractionated radiation) for unresectable locally advanced NSCLC. For the first treatment group, atezolizumab is given 3–4 weeks after completion of chemoradiation for up to 1 year. If the toxicity of this sequential delivery can be tolerated, the second treatment group will be given atezolizumab concurrently with chemoradiation for 6–7 weeks as well as afterwards for up to 1 year. Although this is not a randomized comparison of the two regimens, insights into how the different schedules affect the safety and efficacy of the combined treatment could be useful for future trial design.

### Optimizing the dose of radiotherapy: conventional fractionation or hypofractionation

The radiation dose and fractionation schedule are also important factors to consider when radiation is combined with immunotherapy. The conventional fractionation scheme, that is, 1.8 to 2-Gy fraction given once a day, takes advantage of tumors' vulnerability in terms of DNA repair and cell cycle regulation. However, whether this conventional fractionation schedule, or one that utilizes a hypofractionated schedule (larger doses per day over a shorter course of time to a lower or same total dose) produces the best synergy with immunotherapy remains in question (Figure 1B). Several preclinical studies have compared single high-dose radiation with fractionated radiation for their ability to induce an immune response. In a B16-OVA melanoma model, both single-dose radiation (15 Gy) and fractionated radiation (five fractions of 3 Gy each) delivered to tumors increased the generation of antigen-specific T cells. However, the 15-Gy single-dose regimen generated more tumor-infiltrating T cells than did the fractionated regimen (Lugade et al., 2005). Moreover, the secretion of interferon-γ by cells in tumor-draining lymph nodes was higher in the mice given a single dose than in the mice given fractionated radiation (Lugade et al., 2005). A later report of a B16 mouse model from another group also showed that the immune response triggered by ablative radiation doses was abrogated by conventional fractionation (Lee et al., 2009). In another study of a Lewis lung carcinoma (LLC) murine lung cancer model, 5 fractions of 10 Gy each induced a more robust abscopal effect than 12 fractions of 2 Gy each (Camphausen et al., 2003). However, evidence also exists to show hypofractionation is not favorable when combined with immunotherapy. In preclinical breast cancer models, an abscopal effect was induced only by fractionated radiation, not single-dose radiation, when given in combination with anti-CTLA4 (Dewan et al., 2009). A preclinical study of human prostate cancer cells showed that exposure to multifraction radiation (ten 1-Gy fractions) induced the release of damage-associated molecular pattern molecules more robustly than did single-dose treatment (one 10-Gy fraction) (Aryankalayil et al., 2014). In a recent study, extreme high-dose radiation (20–30 Gy in 1 fraction) was shown to sabotage tumor immunogenicity by inducing DNA exonuclease Trex1 to block cGAS-STING pathway activation (Vanpouille-Box et al., 2017; Ye and Formenti, 2017). In this study, anti-CTLA4 therapy was not able to synergize with high dose radiation to induce an abscopal effect in the TSA mouse mammary carcinoma model. The authors found that the expression of Trex1, a major 3' DNA exonuclease, was significantly upregulated when cells received more than 10 Gy of radiation. As a result, cytosolic DNA was significantly curtailed in cancer cells that received high dose radiation compared to cells that received low dose radiation, and activation of the cGAS-STING pathway to produce interferon- β was greatly attenuated. Knockdown of Trex1 enabled 20 Gy of radiation to induce the abscopal effect with anti-CTLA4 (Vanpouille-Box et al., 2017). Taken together, radiation dose and scheduling appear to be important factors when combined with immunotherapy, and could further be complicated by the types of immunotherapy used. Additional preclinical studies and clinical trials are needed to unravel the optimal radiation dose and scheduling that could best synergize with immunotherapies.

### Minimizing the direct effects of radiation on T cells

The radiosensitivity of T lymphocytes makes them vulnerable targets during radiation therapy. Tumor-infiltrating T cells are inevitably irradiated, especially during prolonged courses of radiation, and the conventional 2-Gy once-daily schedule could be sufficient to inactivate T cells (Deschavanne and Fertil, 1996). These findings raised concern that conventional fractionation may inhibit T cells inside the tumor. In addition to directly irradiating tumor-infiltrating T cells, radiation may also negatively impact T cells in the peripheral blood that transit through the irradiated field (Figure 1C). It has been reported that radiation induces lymphopenia in patients, and that severe lymphopenia was associated with poor prognosis in non-small cell lung cancer and nasopharyngeal cancer (Tang et al., 2014; Cho et al., 2016). We recently found that patients with esophageal cancer had high incidence of grade 4 lymphopenia during chemoradiation therapy that was not apparent when chemotherapy was given alone (Davuluri et al., 2017). Because most immunotherapies depend on functioning T cells, lymphopenia is likely to undermine immunotherapy efficacy. The risk of developing lymphopenia could also be associated with the radiation modality. In the study mentioned above (Davuluri et al., 2017) and a propensity matched follow-up study (Shiraishi et al., 2017), we found proton radiation was significantly associated with reduced grade 4 lymphopenia risk for esophageal cancer patients treated by neoadjuvant or definitive chemoradiotherapy. The dosimetric advantage of proton therapy, which spares surrounding normal tissue from radiation, may be the main reason for the reduced risk of lymphopenia. Whether immunotherapy better synergizes with proton radiation compared with photon therapy needs further investigation. Irradiation of lymph nodes is also an issue to consider, because even though nodal radiation is known to enhance local control in node-positive disease, nodal irradiation presumably would affect immune-specific T cells in the draining lymph nodes. In a preclinical study examing the effects of prophylactic nodal irradiation, mouse models of both colon cancer and melanoma were used to compare tumor growth and tumor-infiltrating lymphocytes after irradiation of just the tumor or the tumor and the draining lymph nodes (Marciscano et al., 2016). The inclusion of the draining lymph nodes in the radiation field did not affect tumor control, but it did reduce the proportion and absolute numbers of tumor-infiltrating CD8^+^ T cells. However, irradiation of the draining lymph nodes increased the levels of T-cell chemoattractants and antigen-specific CD8^+^ cells in the tumor microenvironment (Marciscano et al., 2016), pointing to the complexity of T cell dynamics after nodal irradiation. Whether nodal irradiation synergizes or sabotages checkpoint inhibitors such as anti-CTLA4 or anti-PDL1 remains to be seen, and needs further preclinical and clinical investigation.

### Identifying biomarkers to predict responders to combination therapy

Despite remarkable successes in the past decade, the effectiveness of immunotherapy varies across patients and across cancer types (Nishino et al., 2017). Currently, increasing the response rates to immunotherapy and identifying biomarkers with which to predict which patients are most likely to respond to immunotherapy represent unmet needs for the full implementation of precision immunotherapy (Figure 1D). To date, biomarkers tested for their ability to predict immunotherapy responses include PDL1 expression (Topalian et al., 2012; Herbst et al., 2014; Borghaei et al., 2015; Rizvi et al., 2015), mutational burden and neoantigens (Schumacher and Schreiber, 2015; Shukuya and Carbone, 2016; Balachandran et al., 2017; Khagi et al., 2017; Łuksza et al., 2017), DNA repair deficiencies (Le et al., 2015, 2017), tumor-infiltrating lymphocytes (Daud et al., 2016; Remon et al., 2016), patient HLA class I genotype (Chowell et al., 2017), and serum markers (Ku et al., 2010; Liikanen et al., 2015; Diem et al., 2016; Essink et al., 2016). Recent studies have suggested that the response to immunotherapy could be predicted by the gut microbiome in mouse models (Vétizou et al., 2015) and melanoma patients (Gopalakrishnan et al., 2017). The latter study showed the melanoma patients with more diverse and abundant Ruminococcaceae bacteria responded better to anti-PD-1 therapy. More importantly, the fecal transplant from responding patients to germ-free mice enhanced both systemic and anti-tumor immunity. These findings not only reveal the utility of the gut microbiome as a predictive marker for immunotherapy effectiveness, but also support the notion that immunotherapy response could be altered by the adoptive transfer of the gut microbiome to non-responding patients as a novel therapeutic approach.

Actually, the greatest challenge in predicting response to combination therapy could be that the combination itself may affect the signaling network of the tumor so as to alter its behavior and response to treatment. For example, in preclinical models, the HSP90 inhibitor ganetespib was able to radiosensitize a panel of NSCLC cell lines with diverse genetic backgrounds. However, when tested with chemoradiation, ganetespib could sensitize some cell lines, but not others, both *in vitro* and *in vivo* (Wang et al., 2016). Because predicting response to immunotherapy is itself a challenge, identifying biomarkers to predict outcomes after combined immunotherapy and radiation could be far more challenging. A recent study in which mass cytometry was used to profile immune-cell infiltrates after treatment with either of two checkpoint inhibitors (anti-CTLA4 and anti-PD1) showed that the antitumor effects of each were driven by distinct mechanisms of action (Wei et al., 2017). The responses to immunotherapy also vary across cancer types. A recent genomic analysis of 100,000 human cancers showed the diverse mutation burden across different cancers and the cancer types that had high mutation burdens generally had better responses to immunotherapies, such as melanoma, NSCLC, bladder cancer, and renal cell carcinoma (Chalmers et al., 2017). Because of the complexity of responses for different types of checkpoint blockades and cancers, identifying a “universal marker” that predicts response to all types of checkpoint blockade therapies in all cancer types may not be possible. In addition, several novel checkpoints are emerging in recent years (Torphy et al., 2017), whether radiation synergizes with them still needs to be investigated. Moreover, responses to immunotherapy may emerge later than responses to conventional chemotherapy or other targeted therapies, and thus the criteria and standards for evaluating “response” is still a matter of debate (Nishino et al., 2017).

## In the future: could immunotherapy be a radiation sensitizer?

To date, discussions of synergy between radiation and immunotherapy have focused mostly on how radiation could enhance the therapeutic effects of immunotherapy, as described previously in this current review. However, whether immunotherapy itself could be a radiation sensitizer has not been widely investigated. Radiosensitization agents increase a tumor's sensitivity to radiation, with the promise of enhancing cytotoxicity to the tumor without the need of higher radiation doses. Chemotherapy, monoclonal antibodies, and targeted agents all have radiosensitization effects in several types of tumor (Lawrence et al., 2003; Chen et al., 2004; Milas et al., 2004; Girdhani et al., 2005; Feng et al., 2014; Wang et al., 2016). Indeed, the relationship between radiation and immunotherapy may be more profound and complex than had previously been thought. One might assume that immunotherapy could sensitize tumor cells to radiation on the basis of current knowledge as follows. First, several regulators of both radiosensitivity and immune checkpoints have been identified, among them PARP inhibitors (Alotaibi et al., 2016), which may act by upregulating PDL1 expression and inducing immunosuppression (Jiao et al., 2017). Another well-known radiation response regulator, p53, had also been shown to modulate PDL1 expression (Cortez et al., 2016). Second, immune checkpoint blockade may influence the tumor microenvironment by regulating cytokine secretion (Perrin et al., 1996; Hryniewicz et al., 2006) and by remodeling tumor vasculature (Schoenfeld et al., 2010). Immunotherapy could plausibly affect tumor radiation response through mechanisms that are independent of their effects on immune cells. Given the scarcity of evidence that immunotherapy may have direct or indirect radiosensitizing properties, preclinical and clinical studies will be helpful to ascertain this possibility.

## Conclusion

In summary, radiation seems to synergize with immunotherapy via several mechanisms, such as increasing the visibility of tumor antigens, activating the cGAS-STING pathway, and modulating the tumor microenvironment. Although the combination of radiation and immunotherapy has proven effective in preclinical studies and shows promise in clinical trials (Table 1), challenges still exist for the future application of this combination therapy. The optimization of radiation dose and timing and the identification of potential biomarkers may further enhance the effectiveness of this unique combination. In the meantime, the concept that immunotherapy may act as a radiation sensitizer to improve tumor local control could be another fruitful avenue of investigation.

**Table 1:** Studies using radiotherapy and immunotherapy.

**Study**	**Type**	**RT**	**IT**	**Sequence**	**Results**
Alomari et al., 2016	Case report: brain metastases	SRS 22 Gy	ipilimumab, pembrolizumab	IT, RT, IT	Status improvement
	Case report: brain metastases	SRS 20 Gy	nivolumab, ipilimumab	RT, IT	Remaind asymptomatic neurologically 6 weeks after surgery
Antonia et al., 2017	Stage III trial: lung cancer	Definitive RT (54 to 66 Gy)	durvalumab	RT, IT	PFS improvement with durvalumab
Aryankalayil et al., 2014	Preclinical: human prostate cancer cells	1 Gy × 10 vs. 10 Gy	NA	NA	Multifraction radiation induced more DAMP release
Baird et al., 2016	Preclinical: murine pancreatic	10 Gy	Cyclic dinucleotides	Concurrent	STING activator and RT synergistically controlled local and distant tumors
Camphausen et al., 2003	Preclinical: murine lung (LLC)	10 Gy × 5 vs. 2 Gy × 12	NA	NA	Five fractions of 10 Gy induced more robust abscopal effects
Deng et al., 2014a	Preclinical: murine breast and colon	12 Gy	anti-PD-L1	RT, IT	Combination of radiation and immunotherapy could be more potent than either treatment alone
Dewan et al., 2009	Preclinical: murine breast	20 Gy × 1 vs. 8 Gy × 3 vs. 6 Gy × 5	anti-CTLA4	Concurrent	Abscopal effect was induced only by fractionated radiation
Dovedi et al., 2014	Preclinical: murine melanoma, colorectal and TNBC	10 Gy in 5 fractions	anti-PD-1 or anti-PD-L1	Concurrent, sequential	PD1/PDL1 inhibition was effective only when given either concomitantly with or at the end of radiation
Haymaker et al., 2017	Case report: metastatic melanoma	WBRT 30 Gy in 10 fractions	ipilimumab, pembrolizumab	IT, RT, IT	Status improvement, long-term survival
Lee et al., 2009	Preclinical: murine melanoma (B16)	20 Gy vs. 20 Gy in 4 fractions	NA	NA	Immune response triggered by ablative radiation doses
Lugade et al., 2005	Preclinical: murine melanoma (B16)	15 Gy vs. 15 Gy in 3 fractions	NA	NA	15 Gy single-dose generated more tumor-infiltrating T cells
Nagasaka et al., 2016	Case report: head and neck	Palliative 30 Gy	pembrolizumab	IT, RT	Significant radiographic response
Qian et al., 2016	Clinical: melanoma brain metastasis	SRS 12–24 Gy	anti-CTLA4, anti-PD-1	Concurrent vs. non-concurrent	IT given within 4 weeks of stereotactic radiosurgery led to improved response
Reits et al., 2006	Preclinical: murine colon	10 Gy	T cell adoptive transfer	RT, IT	Combination better inhibited tumor growth
Samstein et al., 2017	Clinical	Various doses	anti-CTLA4, anti-PD-1/PD-L1	Concurrent, non-concurrent	Induction immunotherapy begun more than 30 days before radiation resulted in longer OS
Schoenhals et al., 2016	Case report: lung cancer	Fractionationed RT to primary and metastasis	nivolumab	RT, IT, RT	Abscopal effect
Shaverdian et al., 2017	Stage III trial: lung cancer	Various doses	pembrolizumab	RT, IT vs. IT	Patients who previously received any radiotherapy had better overall survival when treated with pembrolizumab
Shi et al., 2017	Case report: pancreatic cancer	45 Gy in 15 fractions	GM-CSF	Concurrent	Abscopal effect, survival benefit
Twyman-Saint Victor et al., 2015	Preclinical: murine melanoma and pancreatic	20 Gy, 8 Gy	anti-CTLA4, anti-PD-L1	Concurrent, sequential	When combined with radiation, anti-CTLA4 and anti-PD-L1 promotes response through different mechanisms
Vanpouille-Box et al., 2015	Preclinical: murine breast	6 Gy × 5	anti-TGF-beta, anti-PD-1	RT, IT	Anti-PD-1 prolonged survival of mice treated with RT and TGF-beta blockade
Vanpouille-Box et al., 2017	Preclinical: murine breast and colon	8 Gy × 3 vs. 20 Gy	anti-CTLA4	RT, IT	Anti-CTLA4 therapy was not able to synergize with high dose radiation to induce an abscopal effect
Young et al., 2016	Preclinical: murine colon	20 Gy	anti-CTLA4	IT, RT vs. RT, IT	Anti-CTLA4 was most effective when given before the radiation
	Preclinical: murine colon	20 Gy	anti-OX40	IT, RT vs. RT, IT	Anti-OX40 was more effective when given 1 day after the radiation

*SRS, Stereotactic Radiosurgery; WBRT, Whole Brain Radiation Therapy*.

## Author contributions

SL supervised the work. All authors wrote and revised the article.

### Conflict of interest statement

SL has received research funding from Elekta, STCube Pharmaceuticals, Hitachi Chemicals Inc., Peregrine Pharmaceuticals, and Roche/Genentech, has served as consultant for AstraZeneca, and received honoraria from AstraZeneca, US Oncology and ProCure. The other authors declare that the research was conducted in the absence of any commercial or financial relationships that could be construed as a potential conflict of interest.
